# Clinical Efficacy of Acupuncture Combined With Conventional Therapy for C5 Nerve Root Palsy After Posterior Cervical Decompression Surgery: A Randomized Controlled Study

**DOI:** 10.1155/prm/2625552

**Published:** 2025-05-04

**Authors:** Hua Wei, Qingfeng Shen, Yuang Fu, Yubo Tan, Junwei Gao, Yingpeng Xia

**Affiliations:** ^1^Tianjin Union Medical Center, Tianjin Medical University, Tianjin, China; ^2^Department of Spine Surgery, Tianjin Union Medical Center, The First Affiliated Hospital of Nankai University, Tianjin, China; ^3^Tianjin Institute of Spinal Surgery, Tianjin, China

**Keywords:** acupuncture, C5 palsy, cervical, laminectomy, posterior approach, spine surgery

## Abstract

**Objectives:** To observe the clinical efficacy of Tiaokou acupoint-penetrating Chengshan combined with conventional therapy for C5 nerve root palsy after posterior cervical decompression surgery.

**Methods:** A total of 52 patients undergoing posterior cervical decompression surgery at Tianjin Union Medical Center from March 2020 to March 2023 were grouped using computer-generated random numbers. Group A (*n* = 26) received the treatment of acupuncture combined with conventional therapy. Group B (*n* = 26) received only conventional therapy.

**Results:** The VAS scores of the two groups after treatment were significantly lower than scores before treatment (*p* < 0.01). The JOA scores, Lovett muscle strength grade, and shoulder ROM of the two groups after treatment were significantly higher than those before treatment (*p* < 0.01). After treatment, the VAS scores of Group A were significantly lower than those in Group B (*p* < 0.01) while the JOA scores, Lovett muscle strength grade, and shoulder ROM were significantly higher than those in Group B (*p* < 0.01). There were significant differences in VAS difference (*p* < 0.01) and JOA difference (*p* < 0.01) after 2 weeks of intervention treatment in Group A compared to Group B. There was a significant difference in the improvement of shoulder ROM in Group A after 2 weeks of intervention compared to Group B.

**Conclusions:** The acupuncture method of Tiaokou acupoint-penetrating Chengshan combined with conventional nutritional support therapy was more effective in patients with C5 nerve root palsy after posterior cervical decompression surgery compared to conventional nutritional support therapy.

**Trial Registration:** Clinical Trial Registry identifier: ChiCTR2300073583

## 1. Introduction

Cervical 5 nerve root palsy (C5P) is one of the most common complications after posterior cervical decompression surgery, with an incidence reported in the literature ranging from 0% to 30% [1]. The syndrome is characterized by predominantly pain and loss of muscle strength appearing in a delayed fashion in the days or weeks following surgery. The complication was first reported by Scoville and Stoops in 1961 and gradually attracted the attention of clinicians. A large number of studies have been conducted to investigate its incidence and possible causes, and many hypotheses have been put forward, including the theory of dorsal spinal cord migration, spinal cord rotation, preoperative foraminal stenosis, and spinal cord reperfusion injury [2–5]. Relevant mechanisms, however, are still not fully elucidated completely. Most of researchers tend to regard the symptoms as a combination of various factors [6]. In addition, the treatment of C5P is still a matter of concern. Currently, there are no established consensus guidelines or standardized therapeutic protocols for C5P, necessitating reliance on symptomatic management with characteristically slow therapeutic response. We have studied the literature and medical cases and found that the therapy of Tiaokou (ST38) acupoint-penetrating Chengshan (BL57) was very effective in treating shoulder paralysis caused by a deficiency of Yang Qi and a loss of sinew support due to the entry of wind, cold, and dampness [7–12]. Guided by the fundamental TCM principle of “treatment based on syndrome differentiation” (Bianzheng Lunzhi), the therapeutic rationale for employing the ST38-through-BL57 acupuncture method in C5P cases was systematically justified through clinical pattern analysis. Therefore, a prospective study was conducted to investigate the effect of the treatment of the integration of traditional Chinese and Western medicine on C5P, a persistent disease in spinal surgery.

## 2. Methods

### 2.1. Study Design and Randomization

This study was a prospective randomized controlled clinical trial. The Declaration of Helsinki was strictly followed throughout the study. This study was approved by the ethics committee of Tianjin Union Medical Center (2020B07). The voluntary subjects provided written informed consent.

Based on the preliminary pilot study results, the Visual Analog Scale (VAS) score improvement was (4.1 ± 2.0) in the acupuncture group and (2.4 ± 1.9) in the control group. According to the formula: *n*=((*z*_*α*/2_+*z*_*β*_)^2^ × (*σ*_*T*_^2^+*σ*_*C*_^2^)/(*μ*_*T*_ − *μ*_*C*_)^2^), *α* = 0.05 (two-tailed) and *β* = 0.20 (80% power), *n* ≈ 21. Accounting for a 20% dropout rate, the final sample size was adjusted to 26 participants per group, resulting in a total of 52 participants. Participants were randomly distributed with 1:1 allocation ratio using a computer-based program to Group A, which received acupuncture and conventional nutritional nerve support therapy (intervention), and Group B, which received simple conventional nutritional nerve support therapy (control).

The randomization of the patients was performed by an independent researcher, and a nonblinded practitioner physician was informed about the group allocation of the patients. The interventions were performed by the same physician with professional qualification. Outcome measures were assessed at three timepoints: at preoperation, diagnosis, posttreatment-at 2 weeks.These included the VAS, Japanese Orthopedic Association (JOA), recovery of biceps and deltoid muscle strength, range of motion (ROM) of the shoulder joint. Data collection was performed by a single researcher who was blinded to the randomization processes and procedures of patients in each group.

### 2.2. General Information

A total of 52 patients of both sexes aged 40–78 years admitted to our hospital from March 2020 to March 2023 with C5P after cervical decompression were included into this study at the department of spine surgery.

The diagnosis of C5P was made based on the diagnostic criteria widely accepted currently (recent postoperative decline in deltoid and/or biceps muscle strength of more than 1 grade compared to preoperative baseline levels following cervical decompression surgery, without any myelopathic symptoms).

### 2.3. Inclusion Criteria

Patients included in this study who (i) was diagnosed with spinal cervical spondylosis with subsequent first-time posterior cervical decompression; (ii) had postoperative onset of C5P symptoms such as paralysis, pain and loss of muscle strength in the deltoid and/or biceps or triceps muscles; (iii) had no history of shoulder disease before operation and no intraoperative nerve injury; (iv) had normal position of the internal fixation in the neck on postoperative radiographs, without evidence of residual spinal cord compression; (v) had satisfactory incision healing; (vi) had cognitive capacity to comprehend and consent to acupuncture treatment.

### 2.4. Exclusion Criteria

Patients were excluded from this study who (i) did not meet the diagnostic criteria for C5P; (ii) had cervical spine surgery history; (iii) hadconcurrent cervical spinal pathologies (e.g., tumors, infections); (iv) had a history of shoulder complications; (v) had abnormalities such as malpositioned internal cervical fixation on postoperative examination; (vi) rejected acupuncture treatment; (vii) lacked voluntary expression or cognitive ability.

### 2.5. Treatment

In Group A, the patient was treated with the acupuncture treatment of Tiaokou (ST38) acupoint-penetrating Chengshan (BL57) on the contralateral lower limb of the palsy combined with conventional nerve nutrition and dehydration (2 neurotropine intravenous infusions once daily for 7 days and 250 mL glycerol fructose twice daily for 5 days).

**Figure 1:**
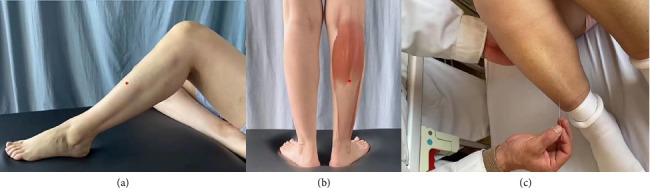
The needling method. (a) The location of Tiaokou (ST38) point (the red point). The point is located eight cun below Dubi (ST35), on the line between Dubi (ST35) and Jiexi (ST41). Dubi (ST35) is located in the depression inferior to the patella and lateral to the patellar ligament. Jiexi (ST41) is located in the central depression in front of the ankle joint, between the extensor hallucis longus tendon and the extensor digitorum longus tendon. (b) The location of Chengshan (BL57) point (the red point). The point is located in the posterior region of the calf, the medial and lateral gastrocnemius muscle belly and muscle-tendon junction. (c) The method of Tiaokou (ST38) acupoint-penetrating Chengshan (BL57).

Acupuncture point: the Tiaokou (ST38) point of the opposite side of the affected limb. Operation: The patient is placed in a supine position with both knees flexed at 90°. Acupuncture was performed using 0.35 mm × 75 mm disposable acupuncture needles. The needle was slowly inserted at an angle of 90° in the Tiaokou (ST38) point with the direction of needle tip to Chengshan (BL57) point, and the stimulation was performed by lifting and twisting for 30 s. Then the needle remained for 30 min. The patient is then helped to move the affected shoulder. The aforementionedinterventionswere administered once every three days, with four sessions constituting a complete treatment course spanning a total duration of 14 days. The needling method is visually illustrated in Figure 1.

In Group B, the patient was treated with conventional neurotrophic and dehydrating treatment (2 neurotropine intravenous infusions once daily for 7 days and 250 mL glycerol fructose twice daily for 5 days).

### 2.6. Outcomes

The average pain severity was measured using a 10 cm VAS scale, with 0 representing “no pain” at one end of the line and 10 representing “the worst pain imaginable” at the opposite end.

JOA scores consist of four parts: upper limb motor function, lower limb motor function, sensation, and bladder function, with a total score of 17 points. The lower the score, the more severe the spinal cord dysfunction.

Manual muscle test Lovett's grading method rates the patient's ability to produce a maximum voluntary contraction of the muscle or muscle group. The test consists of 6 grades (“no disability” = 5, and “full disability”  = 0).

Joint ROM is the maximum arc of motion through which a joint moves, which is the normal ROM of a joint from its beginning to its end.

### 2.7. Statistical Analysis

SPSS 25.0 statistical software was used to process the data. Statistical data were expressed as a chi-square test using the four-compartment table method. Measurement data were expressed as *x* ± *s*. Paired *t*-test was used for comparison before and after intervention within groups for measurement data. Rank data were tested by two-sample rank sum test, and *t*-test and rank sum test were used for comparison of the means of two samples. *p* values less than 0.05 were considered statistically significant.

## 3. Results

### 3.1. General Information About the Patients

As shown in Table 1, there were 22 male cases and 4 female cases in Group A and 18 male cases and 8 female cases in Group B. There were no significant differences in the gender and age between the two groups (*p* > 0.05), so they were comparable at baseline.

### 3.2. Comparison of VAS Scores Between the Two Groups

As shown in Table 2, VAS scores after treatment in group A were significantly lower than the scores before treatment (*p* < 0.01). The same goes for Group B. After treatment, Group A had lower VAS scores than Group B (*p* < 0.01). After 2 weeks of intervention treatment, VAS scores improved more in Group A than that in Group B (*p* < 0.01).

### 3.3. Comparison of JOA Scores Between the Two Groups

As shown in Table 3, JOA scores after treatment in Group A and Group B were significantly greater than the scores before treatment, respectively (*p* < 0.01). After treatment, Group A had greater JOA scores than Group B (*p* < 0.01). There was a significant difference in the JOA scores improvement between the two groups after 2 weeks of intervention treatment, indicating that after treatment, Group A experienced significantly higher JOA scores compared to Group B (*p* < 0.01).

### 3.4. Comparison of the Recovery of Biceps Muscle Strength Between the Two Groups

As shown in Table 4, there was a significant difference in the recovery of biceps muscle strength between the two groups (*p* < 0.05), with Group A outperforming Group B.

### 3.5. Comparison of the Recovery of Deltoid Muscle Strength Between the Two Groups

As shown in Table 5, there was a significant difference in the recovery of deltoid muscle strength between the two groups (*p* < 0.05), with Group A outperforming Group B.

### 3.6. Comparison of ROM of Shoulder Joint Between the Two Groups

As shown in Table 6, the angles of flexion (*p* < 0.01), extension (*p* < 0.05), external rotation (*p* < 0.05), and abduction (*p* < 0.01) were larger in Group A than Group B after treatment.

After 2 weeks of intervention treatment, Group A showed greater improvement than Group B in flexion (*p* < 0.01), extension (*p* < 0.01), internal rotation (*p* < 0.01), external rotation (*p* < 0.01), and abduction (*p* < 0.01).

## 4. Discussion

### 4.1. The Research Status of C5P

C5P, one of the complications of cervical spine surgery, clinically presents with paralysis, pain, and loss of muscle strength in the deltoid and/or biceps or triceps muscles after cervical spine surgery, without worsening of spinal cord disease symptoms. Currently, as for its pathogenesis, it is generally accepted that spinal cord drift leads to nerve root embolism [13]. The rest of the hypotheses, including root artery ischemia, segmental spinal cord change, the length of C5 nerve root bare area were based on the premise of spinal cord drift hypothesis [14]. Due to the lack of specific diagnostic criteria, studies on the incidence, risk factors, prevention, and treatment of C5P after cervical decompression have not been uniformly understood. Maybe the risk of postoperative C5P can be predicted by preoperative imaging measurements. The validity of intraoperative neurophysiological testing remains inconclusive [15, 16]. There is still no consensus on whether to perform a preoperative foraminotomy [17]. Most patients with postoperative C5P can be treated symptomatically with hormones, dehydrating agents, nerve nutrition, and hyperbaric oxygen. In severe cases, intervertebral foramen enlargement, uncovertebral joint resection, and nerve transfer repair can be performed. Previous studies have suggested that the prognosis is good, and most patients returned to normal function by rehabilitation training of weeks to months, but a follow-up study by Nassr et al. found that 54.2% of patients had complete remission, 25.4% had some resolution, and 17.0% had no resolution [18]. Hofler et al. reported a median recovery time of 2 months for patients with C5 palsy after cervical spine surgery, even up to more than 8 months for some patients. 75.5% of patients returned to baseline strength, 86.6% had at least grade 4 strength at the last follow-up, and 7.5% had weaker than baseline strength and less than grade 4 [19]. In conclusion, C5P has seriously compromised the recent outcome of cervical decompression and has also a significant negative impact on patients physically and mentally, so it has become a persistent problem in spinal surgery. Western medicine treatment has no clear standard, and the effect is very slow.

### 4.2. Traditional Chinese Medicine Treatment of C5P

Traditional Chinese medicine implements specific treatment according to symptoms rather than diseases. Although there is no clear definition, C5P, according to the clinical manifestations, should be classified as liver and kidney deficiency, qi stagnation and blood stasis, and obstruction of the meridians, which belong to the category of paralysis. Externally, wind, cold, and dampness arthralgia impede the meridians, resulting in paralysis. Internally, there is a deficiency of qi and blood, a deficiency of liver and kidney, and intraoperative damage to the meridians, which results in poor circulation of qi and blood and stagnation of the meridians, causing pain and movement disorders. The treatment of paralysis in Chinese medicine includes herbal therapy, acupuncture, and manipulation. For C5P that occurred recently after surgery, both herbal and manipulative treatments have low acceptance, so acupuncture is the most feasible option. Jing Du et al. used acupuncture and moxibustion to warm the meridians and free the collateral vessels to treat C5P with remarkable efficacy [20]. However, this approach necessitates the application of acupuncture at multiple points combined with moxibustion therapy, which may increase procedural complexity and treatment duration, which also tends to reduce patient cooperation. Xu et al. used acupuncture point heat-sensitized moxibustion to treat C5P after decompression surgery for spinal cord cervical spondylosis, which effectively relieved the patients' shoulder pain and improved muscle strength, but the moxibustion treatment is inapplicable to postoperative wards [21]. Wang et al. implemented a triple needling protocol targeting specific acupuncture points – Jianyu (LI15), Jianliao (TE14), Jianzhen (SI9), Binao (LI14), Quchi (LI11), Yanglao (SI6), Houxi (SI3), and the affected Ashi points – to address muscle atrophy and pain management. This approach demonstrated clinically observable improvements in both functional recovery and symptom alleviation [22].

In the review of medical cases, we found that the classic method of acupuncture for shoulder paralysis is Tiaokou (ST38) acupoint-penetrating Chengshan (BL57). The Tiaokou (ST38) acupoint, a classical acupoint of the Stomach Meridian of Foot Yangming, corresponds to the Yang Earth in the Five Elements. As Earth governs the extremities, needling Tiaokou activates the Yangming meridian qi and invigorates Yang Qi, thereby facilitating unobstructed circulation of limb meridian qi. Chengshan (BL57), an acupoint on the Bladder Meridian of Foot-Taiyang, serves as a critical anatomical junction for musculoskeletal dynamics, mediating the meridian's regulatory influence on tendon-muscle coordination. Acupuncturing at Tiaokou (ST38) to penetrate the acupuncture point Chengshan (BL57) can unblock the Qi of both Yangming and Taiyang meridians [23]. The Yangming meridians, endowed with an abundant capacity to regulate qi and blood, demonstrate therapeutic potentials in invigorating vital energy, stimulating hematopoiesis, dredging meridians, and unblocking collateral vessels. Taiyang meridians govern the body's exterior, which can expel wind-cold pathogens, dissipate blood stasis, and dredge collaterals. The method of penetrative needling is currently used in traditional Chinese medicine to treat periarthritis of the shoulder. Guo [24] acupunctured 128 cases of periarthritis at the Tiaokou (ST38) point to penetrate Chengshan (BL57), indicating better outcome and shorter course of treatment. Some studies have also shown that the treatment of periarthritis by Tiaokou (ST38) acupoint-penetrating Chengshan (BL57) with local activities was very effective [25]. According to the principle of “syndrome differentiation and treatment” and “treating different diseases with the same treatment,” C5P is categorized as bi syndrome (a traditional Chinese medicine [TCM] disorder characterized by blockage of meridians) in TCM theory. Both conditions may be treated using the penetrating needle technique – a therapeutic approach aimed at dispelling pathogenic wind, dredging obstructed collaterals, and alleviating pain. Tianshi Ye documents that an older woman suffering from Qi deficiency of Yangming and difficulty in flexing and extending the scapula should regulate defense qi and tonify everything in *A Guide to Clinical Practice with Medical Record* [26]. Therefore, if Yang Qi is deficient and the sinews are not nourished, we should warm and tonify Yang Qi by the Foot-Yangming Stomach meridian; if defense-qi is not consolidated and external evil takes advantage of the deficiency, we should warm the meridians, secure the exterior, dispel cold, and relax the sinews by the Bladder meridian. According to *the Plain Questions·Contralateral Acupuncture*, when evil comes to the meridian, the right side will be sick if the left side is strong, and the left side will be sick if the right side is strong. The therapeutic rationale for contralateral acupoint selection stems from the meridian system's bilateral response to pathogenic infiltration. When pathogenic qi invades a meridian, it creates an asymmetrical distribution of meridian qi between body hemispheres, often manifesting symptoms contralateral to the invasion site. Contralateral acupoint strategically intercepts pathogenic progression while restoring bilateral meridian qi homeostasis. Therefore, we choose the contralateral acupuncture point. The patient is helped to move the shoulder at the same time as the acupuncture, combined with conventional dehydration and nerve nutrition treatment to promote recovery of the affected limb.

### 4.3. The Findings and Limitations of This Study

Group A showed significant differences in VAS score, JOA score, biceps and deltoid muscle strength recovery, and shoulder mobility compared with the control group. After 2 weeks of intervention treatment, the VAS difference and JOA difference changed more in Group A than that in Group B. There was a significant difference in the improvement of shoulder flexion, extension, internal rotation, external rotation, and abduction in Group A after 2 weeks of intervention compared to Group B. These findings suggest that acupuncturing Tiaokou (ST38) to penetrate Chengshan (BL57) with conventional therapy can effectively shorten rehabilitation time, improve recovery efficiency, effectively relieve patients' shoulder pain, and restore muscle strength in the shoulder and arm.

Through the randomized controlled trial, we used the integrated therapy of traditional Chinese and Western medicine to treat the persistent disease of C5P after posterior cervical decompression surgery and achieved significant results in just 2 weeks of postoperative hospitalization. However, as few cases were included and no long-term follow-up was conducted, the medium and long-term efficacy of the integrated therapy of traditional Chinese and Western medicine and the rebound of symptoms are yet to be further concluded and verified. In addition, potential confounding factors including heterogeneous patient compliance rates and variability in postoperative rehabilitation protocols were not systematically controlled. Future prospective studies should incorporate standardized telerehabilitation platforms to minimize protocol deviations, while controlling for comorbidity indices through propensity score matching.

## 5. Conclusions

The acupuncture method of Tiaokou (ST38) acupoint-penetrating Chengshan (BL57) combined with conventional nutritional support therapy was more effective, reduced pain, and improved shoulder ROM in patients with C5P after posterior cervical decompression surgery compared to conventional nutritional support therapy.

## Figures and Tables

**Table 1:** General information about the patient.

Group	Gender (%)		Age
	Male	Female	
A (*n* = 26)	22 (84.6)	4 (15.4)	60.27 ± 9.85
B (*n* = 26)	18 (69.2)	8 (30.8)	56.92 ± 9.47
*p*	0.324		0.217

**Table 2:** Comparison of VAS scores between the two groups.

Group	Preoperative	When diagnosing C5P	After 2 weeks of interventional treatment	Improvement	*t*	*p*
A (*n* = 26)	5.42 ± 1.14	5.96 ± 1.46	2.00 ± 0.98^∗∗##^	3.96 ± 1.84^##^	10.957	< 0.01
B (*n* = 26)	5.23 ± 1.53	5.58 ± 1.53	3.50 ± 1.39^∗∗^	2.08 ± 1.13	9.383	< 0.01
*t*	0.541	0.929	−4.491	4.446		
*p*	0.609	0.357	< 0.01	< 0.01		

*Note:* Compared to preintervention, ^∗∗^*p* < 0.01. Compared to Group B, ^##^*p* < 0.01. The improvement was equal to the VAS scores after 2 weeks of intervention minus the scores at the time of diagnosis of C5P.

**Table 3:** Comparison of JOA scores between the two groups.

Group	Preoperative	When diagnosing C5P	After 2 weeks of interventional treatment	Improvement	*t*	*p*
A (*n* = 26)	3.00 ± 1.33	8.42 ± 2.10	12.88 ± 1.51^∗∗##^	4.46 ± 1.61^##^	−15.076	< 0.01
B (*n* = 26)	3.35 ± 1.20	8.12 ± 2.18	10.96 ± 2.13^∗∗^	2.85 ± 1.52	−9.579	< 0.01
*t*	−0.987	0.518	3.765	3.731		
*p*	0.328	0.606	< 0.01	< 0.01		

*Note:* Compared to preintervention, ^∗∗^*p* < 0.01. Compared to Group B, ^##^*p* < 0.01. The improvement was equal to the JOA scores after 2 weeks of intervention minus the scores at the time of diagnosis of C5P.

**Table 4:** Comparison of recovery of biceps muscle strength between the two groups (%).

Group	0	1	2	3	4	5
A (*n* = 26)	0 (0)	0 (0)	1 (3.8)	4 (15.4)	16 (61.5)	5 (19.2)
B (*n* = 26)	0 (0)	3 (11.5)	5 (19.2)	8 (30.8)	8 (30.8)	2 (7.7)
*Z*	10.222					
*p*	0.026					

**Table 5:** Comparison of the recovery of deltoid muscle strength between the two groups (%).

Group	0	1	2	3	4	5
A (*n* = 26)	0	0	2 (7.7)	3 (11.5)	17 (65.4)	4 (15.4)
B (*n* = 26)	0	3 (11.5)	4 (15.4)	9 (34.6)	9 (34.6)	1 (3.8)
*Z*	10.245					
*p*	0.023					

**Table 6:** Comparison of ROM of the shoulder joint between the two groups (°).

	Group	After 2 weeks of interventional treatment	Improvement
Flexion	A (*n* = 26)	110.81 ± 6.86^##^	45.58 ± 13.38^##^
	B (*n* = 26)	89.27 ± 20.42	29.04 ± 20.26

Extension	A (*n* = 26)	39.19 ± 6.87^#^	9.27 ± 6.35^##^
	B (*n* = 26)	35.42 ± 4.73	5.31 ± 3.04

Internal rotation	A (*n* = 26)	31.23 ± 9.87	11.77 ± 7.30^##^
	B (*n* = 26)	27.15 ± 8.39	6.88 ± 4.77

External rotation	A (*n* = 26)	36.08 ± 11.08^#^	15.12 ± 10.24^##^
	B (*n* = 26)	30.58 ± 7.80	5.62 ± 4.61

Outreach	A (*n* = 26)	108.85 ± 17.53^##^	45.54 ± 17.31^##^
	B (*n* = 26)	76.50 ± 14.30	13.96 ± 8.63

*Note:* Compared to Group B, ^#^*p* < 0.05; ^##^*p* < 0.01. The angle improvement was equal to the angle after 2 weeks of intervention minus the angle at the time of diagnosis of C5P.

## Data Availability

The datasets used and analyzed during the current study are available from the corresponding author on reasonable request.
